# Men Who Have Sex with Men in Mozambique: Identifying a Hidden Population at High-risk for HIV

**DOI:** 10.1007/s10461-014-0895-8

**Published:** 2014-09-19

**Authors:** Rassul Nalá, Beverley Cummings, Roberta Horth, Celso Inguane, Marcos Benedetti, Marcos Chissano, Isabel Sathane, Peter Young, Danilo da Silva, Joy Mirjahangir, Mike Grasso, H. Fisher Raymond, Willi McFarland, Tim Lane

**Affiliations:** 1 Ministério da Saúde, Instituto Nacional de Saúde, Av. Eduardo Mondlane/Salvador Allende nº1008, Maputo, Mozambique; 2 Division of Global HIV/AIDS, Centers for Disease Control and Prevention (CDC), Maputo, Mozambique; 3 University of California San Francisco (UCSF), San Francisco, CA USA; 4 International Training and Education Center for Health (I-TECH), Maputo, Mozambique; 5 Pathfinder International, Maputo, Mozambique; 6 Population Services International (PSI), Maputo, Mozambique; 7 Associação Moçambicana de Defesa das Minorias Sexuais (LAMBDA), Maputo, Mozambique

**Keywords:** MSM, HIV, RDS, Seroprevalence, Mozambique

## Abstract

The population of men who have sex with men (MSM) has been largely ignored in HIV-related policies and programming in Mozambique and there is little information about the contribution of MSM to the HIV epidemic. An integrated biological and behavioral study among MSM using respondent-driven sampling was conducted in 2011 in Maputo, Beira and Nampula/Nacala. Men who reported engaging in oral or anal sex with other men in the last 12 months answered a questionnaire and provided a blood sample for HIV testing. The prevalence of HIV was 8.2 % (Maputo, *n* = 496), 9.1 % (Beira, *n* = 584) and 3.1 % (Nampula/Nacala, *n* = 353). Prevalence was higher among MSM ≥ 25 vs. 18–24 years: 33.8 % vs. 2.4 % (*p* < 0.001), 32.1 vs. 2.8 % (*p* < 0.001), and 10.3 vs. 2.7 % (*p* < 0.06), in each city respectively. The difference in prevalence demonstrates the need to increase prevention for younger MSM at risk for HIV and ensure care and treatment for older HIV-infected MSM.

## Resumen

Por lo general, la población de hombres que tienen sexo con hombres (HSH) ha sido ignorada en las normas y programas relacionados con el VIH en Mozambique, y escasea información sobre la contribución de los HSH a la epidemia. Se realizó un estudio biológico y conductual integrado entre HSH usando el muestreo dirigido por los participantes en el 2011 en Maputo, Beira y Nampula/Nacala. Los hombres que informaron haber tenido sexo oral o anal con otros hombres durante los últimos 12 meses contestaron una encuesta y proporcionaron una muestra de sangre para detectar el VIH. La prevalencia del VIH fue del 8.2 % (Maputo, n = 496), 9.1 % (Beira, n = 584) y 3.1 % (Nampula/Nacala, n = 353). La prevalencia era mayor entre los HSH ≥ 25 años de edad vs. 18-24 años de edad: del 33.8 % vs. el 2.4 % (p < 0.001), del 32.1 % vs. el 2.8 % (p < 0.001) y del 10.3 % vs. el 2.7 % (p < 0.06), en cada ciudad respectivamente. La diferencia de prevalencia demuestra la necesidad de aumentar los esfuerzos de prevención dirigidos a hombres jóvenes en riesgo del VIH y de asegurar que los HSH mayores infectados por el VIH reciban cuidados y tratamiento.

## Introduction

An increasing interest in the role of HIV transmission through male-to-male sexual contact in sub-Saharan Africa (SSA) is evidenced by a growing number of research studies seeking to better understand the biological, behavioral and structural factors that affect HIV risk for men who have sex with men (MSM). The data from these studies provide important information about differences among MSM compared to men in the general population in SSA, with HIV prevalence among MSM typically significantly higher [1, 2]. A meta-analysis stratified by geographic region conducted by Baral and colleagues found that MSM were nearly four times as likely to be infected with HIV as men in the general population [3]. In a recent review of 29 studies of predominantly black MSM in SSA countries, HIV prevalence ranged from 7.8 % in a snowball sample conducted in 2007 in Khartoum, Sudan (1.1 % population prevalence) to 49.5 % in a 2008 respondent-driven sample in Johannesburg, South Africa (17.8 % population prevalence) [4]. Factors that have been associated with HIV in MSM throughout SSA vary. There are socio-demographic characteristics such as limited educational attainment, gay identity, and older age that have been associated with HIV infection [4]. The association with older age was also noted in a sample of Ugandan MSM, in which HIV prevalence was significantly higher among MSM more than 25 years old (22.4 %) compared to MSM in the 18–24 year old age group (3.9 %) [5]. Additional behavioral factors correlated with HIV among MSM include frequent unprotected anal sex, limited knowledge of and access to appropriate risk prevention measures, drug use, alcohol use, having regular female partners and, in many settings, commercial/transactional sex [2, 4, 6]. Social and human rights concerns such as discrimination, stigma, violence, and limited access to safe social and health resources are frequently experienced and associated with HIV [2–5].

The accumulating evidence from SSA suggests that MSM in Mozambique may also have a high HIV prevalence and risk behaviors that would make them a key population for targeted HIV prevention, treatment and care services. Mozambique has a generalized HIV epidemic, reportedly driven by heterosexual transmission [7]. Although HIV prevalence appears to be stabilizing, Mozambique has the eighth highest HIV prevalence globally [8, 9]. The national HIV prevalence was 11.5 % in adults in the 15–49 year old age group in 2009 with substantial regional variation [10]. However, little is known about the variation of HIV prevalence in vulnerable key populations. This is particularly true for MSM in Mozambique, who have been largely absent from the national HIV response and priorities [11]. A qualitative study conducted in 2009 in Maputo found that many MSM engaged in HIV risk behaviors and MSM-specific HIV prevention information was scarce. Risk behaviors were similar to those reported in other regional studies and included infrequent condom use with male partners and use of oil-based lubricants during anal sex, increasing the risk of condom breakage [11]. Many MSM in the study believed that anal sex was safer than vaginal sex and that women were the primary source of infection for HIV. The study also noted that some MSM have female partners that foster overlapping sexual networks between MSM and the general population. Additionally, the study described that MSM reported discrimination and hostility from health providers during clinic visits and, as a result, MSM were not likely to visit HIV and STI testing and treatment centers. A modeling exercise exploring modes of transmission used various assumptions to estimate that MSM contribute 5 % of new HIV infections in Mozambique [12]. As is the case with HIV prevalence, there are limited data on specific risks for HIV infection among Mozambican MSM.

As a result of limited data on the biological, behavioral and structural risk factors associated with HIV infection among MSM in Mozambique, the country’s first integrated biological and behavioral survey was conducted from July to November 2011 in order to estimate HIV prevalence and identify behaviors putting Mozambican MSM at risk of HIV infection. The survey used respondent-driven sampling (RDS) to recruit participants. RDS is an approach employed to access relatively hidden and stigmatized populations, which can be hard to reach with traditional survey methods used for the general population [13]. This paper presents key findings from the survey and adds to the scant literature available on MSM in SSA.

## Methodology

### Survey Design

We conducted cross-sectional, integrated biological behavioral surveys (IBBS) at three sites in Mozambique. Prior to survey implementation, we conducted formative research to identify feasibility of RDS, operational needs and potential barriers to participation. Formative research entailed 17 key informant interviews with service providers and stakeholders, and nine focus group discussions with MSM (with approximately 5–10 participants per group). After determining that RDS was the best method for recruiting MSM in Mozambique, we implemented the IBBS in the cities of Maputo, Beira, and Nampula/Nacala. These locations, which represent the three largest metropolitan areas in Mozambique, were selected because formative research found that they had the most extensive MSM networks in the country.

### Study Population

MSM were eligible to participate in the study if they were born biologically male; were 18 years of age or older; had engaged in oral or anal sex with another male in the 12 months preceding the survey; lived, worked or socialized in one of the three areas in the 6 months preceding the survey; possessed a valid referral coupon given to them by a peer; and had not previously participated in the study. Participants provided written informed consent for all study components.

### Sampling

RDS and its theoretical foundations have been described extensively in published literature [13–15]. This systematic chain referral sampling method has been used widely in surveys throughout the world, including SSA [16]. We began RDS in the three areas with the purposeful selection of MSM as “seeds,” based on the size of their social networks and agreed-upon demographic characteristics (e.g., age, educational level, residential area). We used the formative research data to assist with seed selection, which was designed to identify socially well-connected members of the MSM communities. Four seeds initially were selected in each of Maputo, Beira and Nampula cities. These seeds participated in the survey and were subsequently encouraged to refer three MSM from their social networks using study-issued coupons. The MSM recruited by the seeds formed the first wave of recruitment and each of them was instructed to refer three more MSM, and this continued until we observed sample stability (i.e., the point at which the sample composition remained stable across the key demographic and behavioral characteristics) and we approached the target sample size of 500 in each site. In total, we recruited six seeds in Maputo, three in Beira, and eight in Nampula/Nacala. We added two additional seeds in Maputo, the first at 3 weeks and the second at 5 weeks after study initiation, to strengthen recruitment. We revised the sampling plan for the Nampula site by opening a study office and planting five seeds in Nacala, a city that neighbors Nampula and that formative research found has a closely linked social network of MSM despite the 167 km distance between the two cities. Despite this modification, we were unable to meet targeted sample size in Nampula/Nacala; nevertheless, we achieved sample stability on all key variables. Recruitment lasted 18 weeks in Maputo and Beira and 22 weeks in Nampula/Nacala.

### Study Procedures

Potential participants were screened by study staff to ensure eligibility before a trained interviewer administered a computer-assisted interview using Questionnaire Design Studio (QDS 2.6.1) software (Nova Research Company, Bethesda, Maryland). The standardized questionnaire used for the study was adapted from behavioral instruments used in studies of other African MSM and contained several domains, including demographic characteristics, sexual history and condom use, health services access and healthcare seeking behavior, and alcohol and other drug use. Alcohol use was assessed with the AUDIT-C, an adaptation of the Alcohol Use Disorders Identification Test (AUDIT), which is a three question diagnostic tool to help identify people who have hazardous and harmful patterns of alcohol consumption [17]. After completing the interview and receiving pre-test counseling, participants were offered rapid HIV testing and counseling with return of results. In Nampula and Beira, blood collection was by finger prick; in Maputo, venous blood was collected. HIV rapid testing was performed sequentially using the two rapid tests comprising the national HIV testing algorithm in Mozambique. HIV screening was conducted using Determine^®^ HIV-1/2 (Abbott Laboratories, United Kingdom). Non-reactive results were considered negative. Reactive results were confirmed using the Uni-Gold™ *HIV* (Trinity Biotech, Ireland) rapid test. Participants with results reactive to both tests were considered HIV sero-positive and, following post-test counseling, were referred to a nearby health clinic for HIV care and treatment services.

The participants received instructions and three study coupons for referral of other MSM within their social network; during periods of slow recruitment some participants received five coupons in order to stimulate additional recruitment in Maputo and Nampula/Nacala. Participants received an HIV prevention kit, vouchers for mobile phone credit to assist with recruitment and transportation reimbursement as a primary incentive for participating in the study. The estimated value of this incentive was approximately eight US dollars. Participants also received additional vouchers for mobile phone credit valued at approximately two US dollars as a secondary incentive for each eligible peer referred and enrolled in the study. Participants were required to return to receive their secondary incentives. The interview and administrative study procedures were conducted in offices rented specifically for the survey and, due to time and space constraints, in a health center in Nacala.

### HIV Laboratory Testing

The blood collected for HIV rapid testing was also used to prepare dried blood spot cards for centralized HIV testing. The results of the centralized tests were used for surveillance purposes only and results were not returned to participants. We used a sequential testing algorithm with three immunoenzymatic assays (EIA), which detect anti-HIV antibodies. Screening for infection was conducted with Vironostika HIV Uniform II *plus* O (Biomerieux SA, France). Reactive samples were confirmed with Murex HIV 1.2.O (Murex Biotech Limited, Great Britain). Discordant results were retested using Genscreen HIV 1/2 Version 2 (Bio-Rad, France).

### Data Management and Analysis

The data resulting from the interviews were directly entered by the interviewer on a netbook. Coupon tracking data were entered by the coupon manager using RDSCM version 3.0 (Cornell University, Ithaca, New York). The various sources of data were merged into a single database for each site, and each of the three sites was analyzed as a separate survey. The database was verified and cleaned using R version 2.15 (R Core Team, Vienna, Austria) and exported into RDS Analysis Tool (RDSAT version 6.0) (Cornell University, Ithaca, New York). RDSAT was specifically developed for the analysis of RDS data and the software was used to produce population prevalence, 95 % confidence intervals for key variables and calculate survey weights. To make these calculations, RDSAT uses individually reported network size to produce adjusted estimates. Network size was determined by the following questions: “Approximately how many other men who have sex with men do you think live in and around <Study area: Maputo, Beira or Nampula-Nacala> ?”, “Of these, how many do you know by name and they know yours?”, “Of these, how many can you contact in the next month?”, and “Of these, about how many of them would you say are 15 years of age or older?”. The answer to the last question in this cascade was used as the network size question.

RDSAT-produced individual survey weights were exported back to the R statistical package for bivariate analysis using logistic regression (svyglm function) to determine individual associations between HIV prevalence and demographic and risk behavior variables [18]. Variables associated at *p* < 0.02 were used in multivariate analysis. *p* values from Wald tests are reported. *p* values less than 0.05 were considered statistically significant and those between 0.05 and 0.10 as marginally significant. Recruitment network figures were developed using Graphviz 2.30.

### Ethical Approval

The survey was approved by the National Bioethics Committee for Health (*Comité Nacional de Bioética para a Saúde*- CNBS) of Mozambique, the University of California at San Francisco, and the Division of Global HIV/AIDS in the Centers for Disease Control and Prevention, Atlanta.

## Results

A total of 2,595, 1,857 and 2,210 referral coupons were distributed in Maputo, Beira and Nampula/Nacala, respectively; and, of these, 519 (20 %) coupons were returned in Maputo, 727 (39 %) in Beira and 443 (20 %) in Nampula/Nacala. Candidates with coupons were screened for eligibility resulting in enrollment of 496 participants in Maputo, 583 in Beira and 353 in Nampula/Nacala. In Maputo, of the 23 candidates determined to be ineligible, 19 did not meet the study definition of MSM, two had previously participated, one was unable to provide informed consent and one was underage. In Beira, of the 144 ineligible candidates, 89 did not meet the study definition of being MSM, 34 were underage, 12 had previously participated, 5 did not or were unable to provide informed consent and four did not live in the study area. In Nacala, 90 candidates were not eligible to participate; of these, 67 did not meet the study definition of MSM, 14 were underage, four had previously participated, four did not or were unable to consent and one did not live in the study area.

**Fig. 1:**
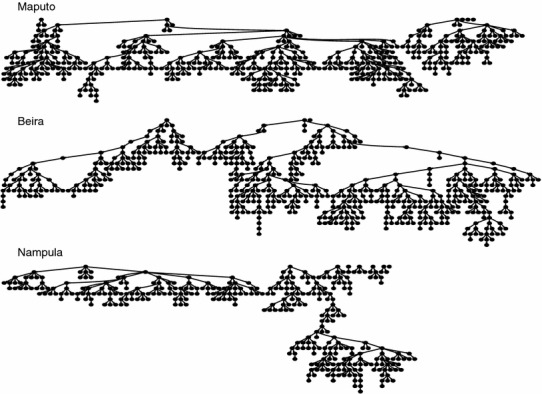
Respondent driven sampling peer-referral recruitment chains of men who have sex with men in the three urban areas, Mozambique, 2011

Results are RDS-adjusted based on six seed participants in Maputo, three in Beira, and eight in Nampula/Nacala. From the six identified seeds in Maputo, three contributed to the recruitment of participants. The longest recruitment chain in Maputo was comprised of 15 waves and 386 participants (77.8 % of the total sample). In Beira, recruitment was achieved through referrals initiated by two of the three seeds. One seed contributed to the recruitment of 444 participants (76.2 % of the total sample) extended across 23 waves. In Nampula/Nacala, two of the eight identified seeds contributed to the recruitment of the majority of the sample with one seed referring 175 (49.6 %) and the other 144 (40.8 %) participants. The maximum number of waves in Nampula/Nacala was 23. Figure 1 illustrates the peer-referral recruitment chains of MSM in the three areas of Mozambique.

Socio-demographic characteristics and circumcision status are presented in Table 1. The median participant age was 22 years in Maputo, and 21 years in both Beira and Nampula/Nacala. Over two-thirds of MSM completed secondary education, although at least 35 % were unemployed in the 12 months preceding the survey. Most MSM (ranging from 86.8 % in Nampula/Nacala to 89.2 % in Beira) had never been married to a woman and few (ranging from 0.4 % in Beira to 8.4 % in Nampula/Nacala) were living in domestic unions with a man (i.e. “living with a man as if married”). In Beira and Nampula/Nacala, 61.3 and 53.5 % of MSM, defined themselves as being either “Gay” or “Homosexual”, while in Maputo 53.1 % self-identified as “Bisexual.” Circumcision status also varied by region and 59.1 % of MSM in Maputo and 97.9 % of MSM Nampula/Nacala were circumcised; while in Beira most MSM (55.9 %) were uncircumcised.

**Table 1:** Socio-demographic characteristics and circumcision status of men who have sex with men in three urban areas, Mozambique, 2011

Variables	Maputo (*N* = 496)			Beira (*N* = 583)			Nampula/Nacala (*N* = 353)		
	*n* ^a^	Adjusted %	(95 % CI)	*n* ^a^	Adjusted %	(95 % CI)	*n* ^a^	Adjusted %	(95 % CI)
Age group									
18–19	176	37.6	(32.0–43.6)	200	42.7	(35.7–50.3)	94	29.4	(22.4–35.5)
20–24	209	44.5	(39.1–49.5)	256	38.8	(33.1–44.0)	199	57.3	(51.1–64.3)
25–29	74	12.1	(8.6–15.5)	92	15.0	(11.2–19.4)	43	10.3	(6.4–14.6)
≧30	37	5.8	(3.5–8.9)	35	3.5	(1.5–6.1)	17	3.1	(1.2–6.1)
Religion									
Christian	404	80.1	(75.0–84.6)	479	82.1	(77.7–86.0)	159	45.4	(38.4–52.4)
Muslim	35	7.9	(4.8–11.4)	45	8.3	(5.4–11.7)	184	53.2	(45.8–60.3)
Other or no religion	57	12.0	(8.4–16.2)	59	9.6	(6.6–12.9)	7	1.5	(0.4–2.9)
Was a student at the time the survey was conducted									
Yes	315	63.5	(58.0–68.5)	418	72.3	(67.0–77.6)	241	76.0	(70.1–81.5)
No	181	36.5	(31.5–42.0)	165	27.7	(22.4–33.0)	109	24.0	(18.5–29.9)
Educational attainment									
None or primary school	73	18.9	(13.7–24.3)	57	10.1	(6.9–13.5)	108	32.2	(24.8–39.3)
Secondary school	383	73.5	(67.7–79.0)	471	84.0	(80.0–87.9)	238	67.6	(60.6–75.1)
Any post-secondary schooling	40	7.6	(4.3–11.3)	55	5.9	(3.7–8.4)	4	0.1	(0.0–0.3)
Was employed in the 12 months preceding the survey									
Yes	327	65.0	(58.8–71.1)	302	45.4	(39.9–51.3)	226	64.2	(57.7–70.2)
No	168	35.0	(28.9–41.2)	281	54.6	(48.7–60.1)	124	35.8	(29.8–42.3)
Marital status (with a woman)									
Never married	428	87.9	(83.8–91.4)	517	89.2	(85.3–93.1)	290	86.8	(81.5–90.6)
Married or living conjugally	32	6.8	(4.0–10.1)	43	7.6	(4.2–11.1)	29	7.4	(4.0–12.1)
Widowed, divorced, or separated	35	5.2	(3.1–8.0)	23	3.2	(1.6–4.9)	31	5.9	(3.8–8.5)
Was married or living conjugally with a man									
Yes	17	2.2	(1.0–3.7)	4	0.4	(0.0–1.0)	27	8.4	(4.6–12.8)
No	479	97.8	(96.3–99.0)	579	99.6	(99.0–100)	323	91.6	(87.2–95.4)
Sexual orientation									
Bisexual	244	53.1	(46.3–59.9)	197	30.0	(24.5–36.1)	113	31.6	(24.7–38.1)
Gay or homosexual	147	20.3	(15.3–26.3)	316	61.3	(54.5–67.4)	200	53.5	(46.2–61.9)
Heterosexual	78	24.3	(17.2–30.2)	32	7.2	(4.1–10.8)	32	14.1	(8.6–19.8)
Other	14	2.3	(1.0–4.5)	8	1.5	(0.5–2.9)	5	0.8	(0.1–1.7)
Circumcision status									
Circumcised	312	59.1	(52.9–65.5)	265	44.1	(38.9–49.4)	342	97.9	(94.9–99.6)
Not circumcised	184	40.9	(34.5–47.1)	318	55.9	(50.6–61.1)	8	2.1	(0.4–5.1)

^a^Categories do not always add up to totals due to missing data

Behavioral characteristics are presented in Table 2. Between 90.7 % of MSM in Maputo and 98.9 % of MSM in Beira engaged in anal sexual intercourse with another man in the 12 months preceding the survey. Thirty-four point nine percent of MSM in Maputo, 43.9 % in Beira and 32.4 % in Nampula/Nacala had more than two male partners in that period, with one in ten MSM in each city reporting more than three male partners. In Maputo and Beira, 86.0 % and 80.3 % of MSM, respectively, used a condom the last time they had anal intercourse with a man; while, in Nampula/Nacala, 61.9 % did so. In Maputo and Beira, 66.6 and 67.7 % of MSM, respectively, used a condom at last vaginal sex with a female partner, while in Nampula/Nacala about half of MSM (49.7 %) did so. Just under half of MSM in Maputo (47.7 %) and 39.2 % in Nampula/Nacala received money, goods, or services in exchange for anal sex with another man in the 12 months preceding the survey. At least 10 % of MSM in each city had a self-reported symptom or diagnoses of an STI in the 12 months preceding the survey. In terms of alcohol consumption, 43.7 % of MSM in Maputo, 43.8 % in Beira, and 32.3 % in Nampula/Nacala were classified as problem drinkers using the AUDIT-C scale and a cut-off ≥4 points. Cannabis use in the 12 months preceding the survey was reported by 12 % of MSM in Maputo and less than 5 % in Beira and Nampula/Nacala; while, less than 3 % of MSM in all cities used other drugs. Two study participants reported having ever injected drugs (not shown).

**Table 2:** Behavioral characteristics of men who have sex with men in three urban areas, Mozambique, 2011

Variables	Maputo (*N* = 496)			Beira (*N* = 583)			Nampula/Nacala (*N* = 353)		
	*n* ^a^	Adjusted %	(95 % CI)	*n* ^a^	Adjusted %	(95 % CI)	*n* ^a^	Adjusted %	(95 % CI)
Ever had anal sex with a man									
Yes, in the last 12 months	461	90.7	(86.6–94.5)	577	98.9	(98.0–99.7)	331	94.3	(91.7–97.8)
Yes, not in the last 12 months	4	1.3	(0.2–2.9)	1	0.1	(0.0–0.5)	2	0.7	(0.0–1.1)
Never	31	8.0	(4.4–12.1)	5	0.9	(0.2–1.9)	17	5.0	(2.0–7.8)
Age of first anal sex with a man									
Never	31	7.4	(3.8–11.1)	5	0.9	(0.1–1.9)	17	4.7	(1.9–7.6)
<15	30	5.2	(2.9–7.7)	75	11.1	(8.0–14.5)	17	5.7	(2.5–10.0)
15–17	125	19.4	(15.5–24.0)	152	27.3	(22.1–32.7)	73	17.9	(13.3–23.0)
18–20	175	41.5	(35.4–48.0)	215	39.3	(33.8–44.6)	153	46.7	(39.0–53.8)
≧21	134	26.5	(20.8–32.8)	132	21.4	(16.7–26.7)	90	25.1	(18.9–32.6)
Total number of male sexual partners (anal sex) in the 12 months preceding the survey									
Never had anal sex with a man	31	7.7	(4.1–11.6)	5	0.9	(0.1–1.8)	17	4.3	(1.7–7.5)
0	4	1.2	(0.1–2.7)	1	0.2	(0.0–0.5)	2	0.5	(0.0–1.1)
1	249	56.2	(50.5–62.2)	301	55.0	(49.5–60.6)	187	62.7	(56.3–68.6)
2	134	23.8	(19.0–28.5)	162	28.6	(23.7–33.6)	102	23.1	(19.1–28.9)
≧3	78	11.1	(8.0–14.6)	114	15.3	(11.6–19.3)	42	9.3	(5.8–12.8)
Offered money, items or services in exchange for anal sex with a man in the 12 months preceding the survey									
No anal sex w/a man in the last 12 months	35	9.4	(5.5–13.7)	6	1.1	(0.3–2.0)	19	5.2	(2.2–8.5)
Yes	40	6.7	(4.3–9.5)	95	14.0	(10.9–17.4)	56	14.6	(9.5–19.3)
No	421	83.9	(79.2–88.3)	481	84.9	(81.4–88.2)	275	80.2	(75.2–86.2)
Received money, items or offered services in exchange for anal sex with a man in the 12 months preceding the survey									
No anal sex w/a man in the last 12 months	35	9.5	(5.6–13.8)	6	1.0	(0.3–2.1)	19	5.0	(2.1–8.5)
Yes	225	47.7	(41.2–54.4)	155	26.5	(21.6–31.6)	147	39.2	(32.2–45.9)
No	235	42.8	(36.3–49.4)	422	72.5	(67.3–77.4)	184	55.7	(49.0–63.1)
Used a condom at last anal sex with a man in the 12 months preceding the survey^b^									
Yes	350	76.0	(70.5–81.1)	471	80.3	(75.8–84.7)	200	61.9	(54.3–68.9)
No	108	24.0	(18.9–29.5)	104	19.7	(15.3–24.2)	128	38.1	(31.1–45.7)
Total number of female sexual partners in the 12 months preceding the survey									
Never had sex with a woman	80	11.4	(8.0–15.9)	169	31.8	(25.6–38.1)	73	15.9	(10.5–22.1)
0	84	12.5	(8.8–16.2)	134	24.8	(19.6–30.0)	69	18.5	(13.2–24.3)
1	107	22.3	(17.5–27.2)	140	24.7	(20.0–29.8)	106	29.2	(22.9–35.3)
2	121	31.5	(25.6–37.7)	51	9.4	(5.9–13.1)	58	19.3	(14.0–25.3)
≧3	104	22.2	(17.5–27.2)	62	9.4	(6.4–13.0)	44	17.1	(11.9–22.8)
Offered money, items or services in exchange for sex (vaginal or anal) with a woman in the 12 months preceding the survey									
No sex w/a woman in the last 12 months	164	25	(19.2–30.2)	303	56.7	(49.7–63.4)	142	34.7	(28.0–42.6)
Yes	85	19	(14.2–24.1)	54	9.2	(5.9–12.6)	73	23.9	(17.9–29.7)
No	247	57	(50.1–63.0)	199	34.1	(28.5–40.2)	135	41.4	(33.7-48.7)
Received money, items or offered services in exchange for sex (vaginal or anal) with a woman in the 12 months preceding the survey									
No sex w/a woman in the last 12 months	164	24.4	(19.2–30.1)	303	56.6	(49.7–63.3)	142	35.4	(28.9–43.6)
Yes	53	10.7	(7.7–14.3)	25	4.6	(2.4–7.2)	37	10.5	(6.9–14.7)
No	279	64.8	(58.9–70.2)	227	38.7	(32.7–45.2)	171	54.2	(46.1–60.5)
Used a condom at last vaginal or anal sex with a woman in the 12 months preceding the survey^b^									
Yes	223	66.6	(60.6–73.7)	166	67.7	(58.5–75.4)	100	49.7	(41.4–59.0)
No	102	33.4	(26.3–39.4)	69	32.3	(24.6–41.5)	99	50.3	(41.0–58.6)
Was diagnosed or had STI symptoms in the 12 months preceding the survey									
Yes	54	10.4	(6.9–14.2)	89	14.4	(11.0–17.9)	45	12.7	(7.6–18.0)
No	442	89.6	(85.8–93.1)	494	85.6	(82.1–89.0)	305	87.3	(82.0–92.4)
Had contact with a peer educator in the 12 months preceding the survey									
Yes	215	40.9	(34.8–47.1)	145	24.3	(19.7–29.6)	176	43.8	(37.1–51.0)
No	279	59.1	(52.9–65.3)	436	75.7	(70.5–80.3)	174	56.2	(49.0–62.9)
Drank alcohol in the last 12 months									
Yes	386	78.2	(72.5–83.1)	376	61.8	(56.2–67.6)	219	59.4	(51.6–66.8)
No	109	21.8	(16.9–27.5)	204	38.2	(32.4–43.8)	131	40.6	(33.2–48.4)
Frequency of alcohol consumption ≧6 drinks/glasses on one single occasion^c^									
Never drinks ≧6	100	28.6	(22.5–36.0)	73	26.3	(20.3–33.5)	81	44.4	(36.1–53.8)
Once a month or less	108	32.3	(25.1–39.6)	67	19.8	(14.4–24.8)	37	15.9	(10.1–21.4)
2–4 times a month	119	32.2	(24.6–40.0)	162	38.2	(32.8–44.6)	28	11.4	(6.7–17.6)
2–3 times a week	18	5.5	(2.7–9.3)	57	12.1	(8.3–15.7)	54	22.2	(14.3–29.7)
4 times or more a week	5	1.4	(0.1–2.2)	11	1.3	(0.4–2.4)	19	6.1	(3.1–9.7)
Consumed alcohol in a manner indicative of probable abuse and/or alcohol dependency (AUDIT-C)									
Yes	257	43.7	(36.7–51.5)	289	43.8	(37.3–49.1)	140	32.3	(25.0–39.1)
No	198	56.3	(48.5–63.3)	277	56.2	(50.9–62.7)	210	67.7	(60.9–75.0)
Used drugs in the 12 months preceding the survey									
Yes	78	15.0	(11.0–19.2)	33	4.7	(2.7–6.9)	28	5.5	(2.7–8.3)
No	418	85.0	(80.8–89.0)	550	95.3	(93.1–97.3)	322	94.5	(91.7–97.3)
Type of drug used in the 12 months preceding the survey									
Cannabis	61	11.8	(8.2–15.7)	29	4.3	(2.4–6.6)	21	4.0	(1.6–6.7)
Other	15	3.1	(1.2–5.2)	3	0.3	(0.0–0.7)	7	1.6	(0.4–3.0)
Never used drugs	418	85.1	(80.8–89.2)	550	95.4	(93.1–97.4)	322	94.4	(91.4–97.2)

^a^Categories do not always add up to totals due to missing data

^b^Includes only MSM who had anal sex with a man in the last 12 months

^c^Includes only MSM who drank alcohol in the last 12 months

**Fig. 2:**
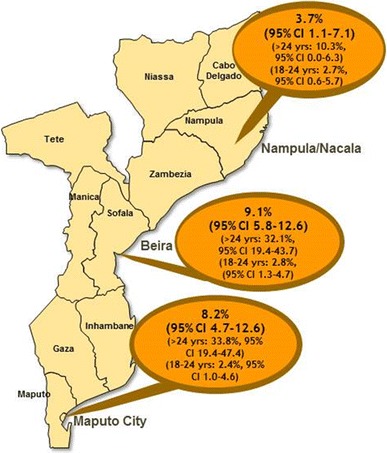
HIV Prevalence among men that have sex with men in three urban areas*, Mozambique, 2011. *2009 estimates of HIV prevalence for the general population were 16.8 % for Maputo City, 15.5 % for Sofala Province, and 4.6 % for Nampula Province

HIV prevalence among MSM (Fig. 2) was 8.2 % [95 % Confidence Interval (CI) 4.7–12.6] in Maputo, 9.1 % (95 % CI 5.8–12.6) in Beira, and 3.7 % (95 % CI 1.1–7.1) in Nampula/Nacala. Prevalence among MSM 25 years of age and older was 33.8 % (95 % CI 19.4–47.4) in Maputo, 32.1 % (95 % CI 19.4–43.7) in Beira and 10.3 % (95 % CI 0.0–26.3) in Nampula/Nacala and is significantly higher than the prevalence among MSM between the ages of 18–24 years (Table 3).

**Table 3:** Association between risk factors and HIV infection among men who have sex with men in three urban areas, Mozambique, 2011

	Maputo					Beira		Nampula/Nacala		
	Crude *n*/*N*	Adjusted %		(95 % CI)	Crude *n*/*N*	Adjusted %	(95 % CI)	Crude *n*/*N*	Adjusted %	(95 % CI)
Age groups										
18–24^a^	14/344	2.4	(1.0–4.6)		15/454	2.8	(1.3–4.7)	7/292	2.7	(0.6–5.7)
≥25	36/103	33.8	(19.4–47.4)^b^		38/127	32.1	(19.4–43.7)^b^	4/59	10.3	(0.0–26.3)^c^
Primary language spoken at home										
Portuguese^a^	32/353	5.6	(2.6–9.2)		28/343	7.2	(3.9–11.1)	8/246	3.5	(0.4–8.8)
Other	18/94	15.1	(6.4–27.0)^b^		25/238	11.6	(6.0–17.7)	3/102	2.5	(0.0–5.8)
Religion										
Christian^a^	44/368	8.1	(4.6–12.6)		37/478	8.7	(5.1–12.4)	6/158	3.2	(0.6–6.6)
Muslim	4/31	4.2	(0.0–11.6)		7/44	14.2	(4.4–27.0)	4/183	3.0	(0.0–10.3)
Other/none	2/48	7.0	(0.0–21.5)		9/59	8.0	(2.9–14.5)	NS^d^	–	–
Level of education										
None or primary^a^	17/67	16.9	(6.3–30.8)		15/57	27.0	(9.7–40.9)	2/108	1.2	(0.0–3.5)
Secondary and above	33/380	5.7	(2.6–9.4)^b^		38/524	7.2	(4.6–10.2)^b^	9/240	4.5	(1.1–9.7)
Marital status w/a woman										
Never married^a^	32/386	6.4	(3.0–9.8)		33/516	5.9	(3.5–8.8)	7/289	3.6	(0.6–7.1)
Married or living conjugally	13/29	30.5	(9.6–55.3)^b^		13/42	38.0	(12.6–58.8)^b^	1/28	2.0	(0.0–9.1)
Widowed, divorced or separated	5/31	17.0	(0.0–36.2)		7/23	20.4	(4.6–39.0)^b^	3/31	6.3	(0.0–26.5)
Condom use at last anal sex with a man in the 12 months preceding the survey										
Yes^a^	25/312	4.4	(1.4–7.4)		42/469	8.6	(5.2–12.2)	7/198	4.8	(0.8–9.8)
No	19/99	17.4	(8.8–28.4)^b^		11/104	9.7	(3.4–17.0)	3/128	3.0	(0.0–9.6)
Condom use at last sex (vaginal or anal) with a woman in the 12 months preceding the survey										
Yes^a^	13/203	5.8	(1.7–10.5)		13/165	7.1	(2.1–14.7)	3/98	2.7	(0.0–6.6)
No	17/97	13.7	(6.2–23.7)^b^		10/69	20.8	(5.2–38.9)^b^	1/99	0	(0.0–0.0)
Had contact with a peer educator in the 12 months preceding the survey										
Yes	15/194	3.7	(1.3–6.7)		16/143	16.7	(8.2–26.0)	6/174	3.4	(0.0–9.4)
No	35/251	11.1	(6.2–17.3)^b^		37/436	6.6	(3.9–9.6)^b^	5/174	3.3	(0.4–7.7)
Gave money, an item or a service in exchange for sex (vaginal or anal) with a woman in the 12 months preceding the survey										
Yes	8/81	9.3	(2.2–18.9)		10/54	18.7	(6.4–34.7)	4/72	3.6	(0.0–9.6)
vNo^a^	23/226	8.4	(3.9–14.0)		14/198	9.9	(3.5–17.2)	NS^d^	–	–
Received money, an item or a service in exchange for anal sex with a man in the 12 months preceding the survey										
Yes	22/198	9.6	(4.1–17.2)		21/155	13.2	(6.9–20.0)	5/147	7.8	(1.3–16.2)
No	23/215	6.7	(2.8–11.8)		32/420	7.4	(3.9–11.3)	5/182	1.7	(0.0–3.0)^c^
Had an STI symptom or diagnosis in the 12 months preceding the survey										
Yes^a^	15/53	22.2	(8.7–41.4)		16/87	15.3	(7.6–24.4)	3/45	10.4	(0.0–28.3)
No	35/394	6.4	(3.3–10.3)^b^		37/494	7.9	(4.5–11.6)^c^	8/303	2.6	(0.5–6.2)

*NS* Not shown due to *N* < 20

^a^Logistic regression reference category

^b^Logistic regression Wald test *p* value: < 0.05

^c^Logistic regression Wald test *p* value: 0.05 ≤ *p*≤0.1

In bivariate analysis (Table 3), HIV prevalence was significantly higher among MSM in Maputo and Beira with no or primary level of education compared to those with secondary level of education and above [16.9 % (95 % CI 6.3–30.8) vs 5.7 % (95 % CI 2.6–9.4), respectively, in Maputo and 27.0 % (95 % CI 9.7–40.9) vs 7.2 % (95 % CI 4.6–10.2) in Beira]. In those same cities, prevalence also differed between MSM who were currently married or in a domestic union with a woman and those who had never been married to a woman [30.5 % (95 % CI 9.6–55.3) vs 6.4 % (95 % CI 3.0–9.8) in Maputo and 38.0 % (95 % CI 12.6–58.8) vs 5.9 % (95 % CI 3.5–8.8) in Beira]. MSM in Maputo and Beira who had not used a condom at last sex with their most recent female partner had higher HIV prevalence than those who had used a condom [13.7 % (95 % CI 6.2–23.7) vs 5.8 % (95 % CI 1.7–10.5) in Maputo and 20.8 % (95 % CI 5.2–38.9) vs 7.1 % in Beira (95 % CI 2.1–14.7)]. In Maputo, prevalence was higher among MSM whose primary language was not Portuguese than among those who spoke primarily Portuguese at home [15.1 % (95 % CI 6.4–27.0) vs 5.6 % (95 % CI 2.6–9.2)], among MSM who did not use a condom at last anal sex with a male partner compared to those who did [17.4 % (95 % CI 8.8–28.4) vs 4.4 % (95 % CI 1.4–7.4)], and among MSM who had STI symptoms or diagnosis versus those that did not have one [22.2 % (95 % CI 8.7–41.4) vs 6.4 % (95 % CI 3.3–10.3)].

In multivariable analysis (Table 4), the odds of having HIV increased significantly with age [adjusted odds ratio (AOR) 1.30 (95 % CI 1.18–1.53) in Maputo, AOR 1.30 (1.22–1.40) in Beira and AOR 1.25 (1.14–1.38) in Nampula/Nacala]. In Maputo, the odds of having HIV was greater among MSM who had STI symptoms or diagnosis in the 12 months preceding the survey than among those who did not have one (AOR 4.06, 95 % CI 1.58–10.46), while the odds was lower among MSM who had contact with a peer HIV educator than among those who did not (AOR 0.18, 95 % CI 0.06–0.55). In Nampula/Nacala, the odds of having HIV was higher among MSM who received money or goods in exchange for anal sex with a man in the 12 months preceding the survey than among those who had not (AOR 5.36, 95 % CI 1.21–23.66).

**Table 4:** Independent associations of HIV infection among MSM in Maputo, Beira and Nampula, Mozambique, 2011

Variable	Maputo AOR^a^ (95 % CI), *p* value	Beira AOR (95 % CI), *p* value	Nampula AOR (95 % CI), *p* value
Age (per year)	1.30 (1.18–1.53), 0.001	1.30 (1.22–1.40), <0.001	1.25 (1.14–1.38), <0.001
Received money, items or services in exchange for anal sex with a man in the 12 months preceding the survey	NS^b^	NS	5.36 (1.21–23.66), 0.03
Peer education contact	0.18 (0.06–0.55), 0.002	NS	NS
Had an STI symptom or diagnosis in the 12 months preceding the survey	4.06 (1.58–10.46), 0.004	NS	NS

*NS* not significant

^a^Adjusted odds ratio (AOR): effects adjusted for other variables listed and weighted for the RDS survey design

## Discussion

HIV prevalence among MSM is relatively consistent with that in the general male population in Mozambique. However, our multivariable analysis showed a dramatic increase in the odds of HIV infection among MSM aged 25 years and older compared to both younger MSM and men of comparable age in the general population. Such dramatic differences by age have been observed throughout the SSA region, and are consistent with evidence that MSM in SSA 25 years and older are at substantially higher risk for HIV infection than the general population [2, 19, 20]. We draw two important conclusions from our prevalence findings. First, there is an urgent need to ensure access to MSM-tailored HIV treatment and care services as part of comprehensive positive health, dignity and prevention (PHDP) programming, particularly for older, seropositive MSM. The PHDP framework advocates for a complete and healthy life for people living with HIV while promoting the reduced risk of HIV transmission to others through strategies such as adherence and assessment of partner status and referrals for partner testing [21]. These strategies are fundamental for stopping onward HIV transmission and for addressing HIV within this population. Second, the relatively low HIV prevalence observed among younger MSM in three of Mozambique’s largest urban areas presents an important opportunity to scale up effective HIV prevention programming for this group. Timely implementation of behavioral and social marketing interventions, as recommended by WHO guidelines, could be helpful in sustaining these encouraging trends and will help to prevent an expansion of the HIV epidemic in this population [22]. For younger Mozambican MSM in particular, it may be advantageous to adopt HIV prevention interventions that capitalize on the growing use of the internet and social media for added relevance and to explore the feasibility of new bio-medical prevention such as pre-exposure prophylaxis and rectal microbicides in the country.

Regardless of serostatus or age, our study findings reveal the need to improve additional core HIV prevention activities. We observed relatively high rates of consistent condom use with male partners, but less with female partners in all three cities. Strengthened efforts for consistent condom use to reduce the frequency of unprotected sex with both male and female partners are needed, including information and risk reduction counseling specifically tailored for MSM. Our study also found that the odds of having HIV was greater among MSM who had an STI symptom or diagnosis in the 12 months preceding the survey in Maputo. This finding, in combination with the increased risk of HIV acquisition posed by STI, warrants enhanced STI screening for MSM in Mozambique.

Finally, a substantial proportion of MSM reported engaging in anal transactional sexual relationships with other men, and in Nampula/Nacala we observed a significant 5-fold odds of HIV infection among those reporting anal transactional sex. We also observed significant associations between lower education levels and higher HIV prevalence in bivariate analysis. Although these associations were not sustained in multivariable analysis, we nonetheless recommend that HIV programming for MSM must explicitly address the potential role that such social and structural factors play both in risk behavior and in accessing HIV prevention, testing, and treatment services. One intervention that has been used successfully in the United States and is being adapted in the region is the Mpowerment Project, which mobilizes MSM to shape a healthy community, cultivate positive social connections and support safer sex practices [23]. The guiding principles of this particular intervention focus on addressing HIV prevention within the context of social issues and community empowerment. This intervention, or a similar approach, would be one avenue for beginning to address these challenging structural issues.

Although alcohol use was not associated with HIV infection in this survey, we estimate that more than one-third of all MSM in each city are excessive alcohol consumers. Alcohol abuse and dependency is a serious public health problem in its own right; furthermore, despite the absence of statistical association with HIV in this study, qualitative studies of MSM communities in the SSA region note that MSM are themselves keenly aware of the relationship between excessive alcohol use and inconsistent condom use [24, 25], and it has been shown to be associated with HIV risk behavior endpoints [24]. Prevention interventions should seek to address the role of alcohol use in sexual behavior and inconsistent condom use among men.

The study has several limitations. Social desirability and interviewer bias may have been introduced by using interviewer-administered questionnaires. Although the interviewers were trained specifically for this study to be objective and avoid judgmental attitudes, our results are possibly skewed given the sensitive nature of sexual behavior questions. Additionally, MSM of younger age and higher education levels are overrepresented in our crude sample. Although RDS adjustment is designed to correct for the effects of recruitment bias, it is possible that our adjusted results retain some bias towards the characteristics of these groups. It is therefore possible that our results underestimate the real prevalence of HIV and associated risk behaviors. In addition, distance and transportation costs between the city of Nampula and the port city of Nacala, 100 km away, presented a significant structural barrier to participation for MSM who resided in Nacala. The extent of this barrier was apparent only after several months. Although we opened a study office in Nacala in October 2011, this allowed for only 6 weeks of recruitment for Nacala participants. It is therefore possible that our Nampula/Nacala sample did not have sufficient time to diversify and results for this city may be further biased towards the characteristics of those participants most easily able to access the study office. Finally, we present our results with full awareness that there is debate over whether theoretical assumptions about recruitment patterns and design effects are met under real-world conditions, and whether multivariable analysis is meaningful under these conditions [26]. Nonetheless, our results are the product of rigorously conducted RDS recruitment and data analysis procedures as they currently exist, and we are confident that our prevalence estimates provide a meaningful reflection of the reality of the HIV epidemic in the MSM population in each area.

Overall, the results of our study point to the need to enhance MSM-tailored programming in Mozambique. Homosexuality is not explicitly illegal in Mozambique and, as a result, barriers to working directly with MSM may be less difficult to overcome than in some other SSA countries. This opportunity should be capitalized upon to expand community-based HIV prevention efforts with MSM, including peer education and social marketing of HIV testing, and condoms. Critical to the success of these efforts will be to have a better understanding of the more complex social and structural factors that can affect scale-up of these interventions and their ability to achieve their intended objectives, including, education, poverty, and alcohol use. This may require additional operations and implementation research in order to adapt evidence-based intervention approaches to the local context of Mozambique. Additionally, it is imperative to include Mozambican MSM in national HIV/AIDS strategic planning for prevention, testing, and treatment interventions. Collaborative strategies that include the participation of MSM, LGBT advocates, healthcare professionals and particularly the public and political powers may ensure the greatest access to and benefit from HIV prevention and assistance interventions directed at this vulnerable population.
